# DOES PARTICIPATING IN SUPPLEMENTAL NUTRITION ASSISTANCE PROGRAM IMPROVE THE SLEEP OF OLDER ADULTS?

**DOI:** 10.1093/geroni/igz038.526

**Published:** 2019-11-08

**Authors:** Queendaleen C Chukwurah, M Pia Chaparro

**Affiliations:** 1 Tulane Center for Aging, Tulane University, New Orleans, Louisiana, United States; 2 Department of Global Community Health Behavioral Sciences, Tulane University, New Orleans, Louisiana, United States

## Abstract

Food insecurity among lower income adults is associated with adverse health outcomes including sleep disorders. Using data from the National Health and Nutrition Examination Survey (NHANES), 2005-2008, we explored the association between participation in the Supplemental Nutrition Assistance Program (SNAP), a safety net program addressing food insecurity, and sleep outcomes among older adults. Total sleep duration (<7 hours inadequate sleep, ≥7 hours adequate sleep) and sleep latency (10-20 mins normal sleep latency, ≤ 9 mins and ≥ 21mins abnormal sleep latency) were available for 805 participants 50 years and older and eligible for SNAP participation (≤130% federal poverty level). SNAP participation (yes/no) was assessed for the previous year. Sleep inadequacy was higher among SNAP participants (46.5%) compared to SNAP-eligible non-participants (36.3%), whereas the corresponding numbers for abnormal sleep latency were 71.8% and 64.1%. Binary logistic regression analysis showed no significant association between SNAP participation and inadequate sleep duration (OR=1.19, 95% CI 0.84-1.70) after adjusting for age, gender, race/ethnicity, education, and food security status. SNAP participation was also not associated with abnormal sleep latency (adjusted OR=1.49, 95% CI 1.00-2.23). SNAP participation was not protective against poor sleep outcomes among a sample of low-income, at risk of food insecurity older adults.

